# Remission in schizophrenia: results of cross-sectional with 6-month follow-up period and 1-year observational therapeutic studies in an outpatient population

**DOI:** 10.1186/1744-859X-11-1

**Published:** 2012-01-05

**Authors:** Sergey N Mosolov, Andrey V Potapov, Uriy V Ushakov

**Affiliations:** 1 Department for Treatment of Mental Disorders, Moscow Research Institute of Psychiatry, Moscow, Russia; 2 Moscow Outpatient Psychiatric Service 21, Moscow, Russia

## Abstract

**Background:**

A standardized definition of remission criteria in schizophrenia was proposed by the International group of NC Andreasen in 2005 (low symptom threshold for the eight core Positive and Negative Syndrome Scale (PANSS) symptoms for at least 6 consecutive months).

**Methods:**

A cross-sectional study of remission rate, using a 6-month follow-up to assess symptomatic stability, was conducted in two healthcare districts (first and second) of an outpatient psychiatric service in Moscow. The key inclusion criteria were outpatients with an *International Classification of Diseases*, 10th edition (ICD-10) diagnosis of schizophrenia or schizoaffective disorder. Remission was assessed using modern criteria (severity and time criteria), PANSS and Global Assessment of Functioning (GAF). Patients who were stable but did not satisfied the symptomatic criteria were included in a further 1-year observational study, with the first group (first district) receiving risperidone (long-acting, injectable) (RLAI) and the second group (second district) continuing to receiving routine treatment. Symptoms were assessed with PANSS, social functioning with the personal and social performance scale, compliance with rating of medication influences scale, and extrapyramidal side effects with the Simpson-Angus scale.

**Results:**

Only 64 (31.5%) of 203 outpatients met the criteria for symptomatic remission in the cross-sectional study, but at the end of the 6-month follow-up period, 158 (77.8%) were stable (irrespective of remission status). Among these only 53 (26.1%) patients fulfilled the remission criteria. The observational study had 42 stable patients in the RLAI group and 35 in the routine treatment group: 19.0% in the RLAI group and 5.7% in the control group met remission criteria after 12 months of therapy. Furthermore, reduction of PANSS total and subscale scores, as well as improvement in social functioning, was more significant in the first group.

**Conclusions:**

Only around one-quarter of our outpatient schizophrenic population met full remission criteria. Use of RLAI gave a better remission rate than achieved in standard care with routine treatment. Criteria for remission should take into account clinical course and functioning to support clinical care.

## Introduction

Remission is commonly used in clinical practice to describe the stable state of schizophrenic patients. Modern classifications such as the *Diagnostic and Statistical Manual of Mental Disorders*, fourth edition (DSM-IV [1]) and the *International Classification of Diseases*, 10th edition (ICD-10 [2]) split remission into full and partial, based on residual symptom occurrence, which gives a lack of symptomatic and functional definitions of status, and, consequently, an absence of specific defined therapeutic targets within the systems; therefore the diagnosis of remission becomes highly subjective. Current clinical psychiatry requires effective methodology to assess therapeutic interventions, which, in turn, necessitate standardized criteria for remission.

The first international consensus-based criteria, proposed by the Remission in Schizophrenia Working Group [3], include two factors: a symptomatic (low threshold of the eight core Positive and Negative Syndrome Scale (PANSS) symptoms [4]) criterion and a time/duration (6 consecutive months) criterion. Selected symptoms based on three dimensions (positive, negative, disorganization), present distinct components for the disorder and incorporate five diagnostic criteria for schizophrenia as specified in DSM-IV.

Validation of the international remission criteria was made in naturalistic [5-7] and intervention [8,9] studies. De Hert *et al*. [5] evaluated the maintenance of remission over 1 year in a large naturalistic prospective study: 99 (29%) of the 341 patients met full remission criteria at the end point, whereas 147 (43%) patients did not meet the symptomatic criterion. Patients in remission had better insight of their disorder and global and daily living functioning. In a 12-week non-interventional naturalistic study with quetiapine, 526 (59%) of 893 outpatients achieved symptomatic remission [7]. Furthermore, analysis of pooled data from 52-week, randomized, double-blind, multicenter comparative trials of 1,283 acute patients showed both symptomatic and time criterion of remission in 32% subjects treated with aripiprazole and 22% with haloperidol reach [8].

### Aims of the study

The primary objectives of this cross-sectional study with a 6-month follow-up period were to evaluate the presence of schizophrenia patients in remission, and to assess maintenance of remission status and symptomatic stability over 6 months in a population from a standard state psychiatric outpatient service in Moscow, Russia.

The primary objective of the 1-year observational therapeutic study was to assess the achievement of remission criteria in a sample of stable schizophrenia patients who did not satisfy symptomatic criterion for remission after a 6-month period and to compare two naturalistic pharmacotherapeutic approaches: routine (mostly first generation antipsychotics) and contemporary (risperidone long-acting, injectable) (RLAI), recently approved as a part of the state reimbursement program) in two comparable city district populations of schizophrenic outpatients.

The secondary objectives of these studies were (1) to assess the achievement of symptomatic remission in different clinical types/courses of schizophrenia according to the ICD-10 diagnostic manual, (2) to detect the most significant factors associated with the achievement of remission in the sample of clinically stable patients, and (3) to assess the prevalence of antipsychotic medications routinely used in Moscow outpatient settings for the long-term treatment of schizophrenia patients (pharmacoepidemiological study).

## Methods

### Design and patients

The cross-sectional study of remission rate was conducted in two healthcare districts (first and second) of the state psychiatric outpatient service. We included outpatients with ICD-10 diagnoses of schizophrenia (F20.0, F20.1, F20.2, F20.3, F20.5, F20.6) and schizoaffective disorder (F25). Patients with diagnoses F20.8 (other schizophrenia) and F20.9 (schizophrenia, unspecified) and other psychotic, schizotypal, and delusional disorders were excluded. The ICD-10 diagnosis was determined via the Mini-International Neuropsychiatric Interview (MINI) [9], plus analysis of case report forms and psychiatric histories from the patients and their families. The type of previous antipsychotic treatment was registered (pharmacoepidemiological study). Nightly administration of antipsychotic drugs at non-therapeutic dosage levels was not considered as an antipsychotic treatment. Patients who did not meet symptomatic criterion for remission were prospectively observed over 6 months for the assessment of stability (no change of PANSS total score > 20% and/or > 1 point for any item of the positive subscale PANSS (P1, P2, P3, P6) regardless of baseline rating). Patients in symptomatic remission were observed for 6 months to assess maintenance of remission status. Stable patients were stratified as having remission and non-remission status, according to the ICD-10 diagnosis.

In the second stage, all stable patients who did not meet symptomatic criterion for remission were offered the option to continue participation in a 1-year observational study. In the first district (first group), patients were offered the option to switch from their current antipsychotic medication to long-acting injectable risperidone (RLAI), while patients in the second district were offered the option to participate in the observational study and continue to receive routine naturalistic treatment (second group).

Data in both studies were collected during routine visits after obtaining written consent from participants (separately for cross-sectional and observational studies).

### Medications in the observational study

The medication used in the observational study was within the state reimbursement program and was administered by a district psychiatrist. In the first group of 42 patients, intramuscular injections of RLAI (25, 37.5, or 50 mg) were administered every 2 weeks. Other antipsychotics were forbidden; only 2 or 4 mg oral risperidone was allowed for titration or in cases of psychotic symptom exacerbation.

In the second group of 35 patients, antipsychotic treatment was routine: 5 (14.3%) patients took a monotherapy of atypical antipsychotic drugs (2 oral risperidone, 2 clozapine, 1 quetiapine), 24 (68.6%) took a monotherapy of typical antipsychotics (4 fluphenazine decanoate, 5 haloperidol decanoate, 7 haloperidol, 3 trifluoperazine, 2 zuclopenthixol decanoate, 2 flupentixol decanoate, 1 chlorpromazine), and 6 (17.1%) took combined therapy (1 fluphenazine decanoate and clozapine, 1 haloperidol decanoate and chlorpromazine, 2 oral haloperidol and chlorpromazine, 1 zuclopenthixol decanoate and trifluoperazine, and 1 oral risperidone and trifluoperazine).

In both groups allowable medication included anti-parkinsonian (anticholinergic) drugs, antidepressants, mood stabilizers, hypnotics, and benzodiazepines for reduction of agitation.

### Assessment

We defined remission according to PANSS operational criteria [4] set up by the Remission in Schizophrenia Working Group [3]. The symptomatic criterion includes eight core PANSS items (delusion, unusual thought content, hallucinatory behavior, conceptual disorganization, mannerism/posturing, blunted affect, social withdrawal, lack of spontaneity) with a score ≤ 3. The duration criterion is symptomatic remission maintenance over 6 consecutive months.

In the cross-sectional study, a homogeneous cohort of outpatients were assessed with the symptomatic criterion of remission, the validated Russian language version of the PANSS [10] and the Global Assessment of Functioning (GAF) scale [1].

In the observational study, we assessed symptom severity with PANSS [10], social functioning with the Personal and Social Performance (PSP) scale [11] and compliance with the Rating of Medication Influences (ROMI) scale (compliance and non-compliance subscales) [12]. All adverse events during the study were recorded. Extrapyramidal side effect rates were assessed with the Simpson-Angus scale (SAS) [13]. Body weight gain was considered to be a 7% increase compared to baseline. In the RLAI group, assessments of quality of life (Short-Form 36 (SF-36) Health Survey [14]) and cognitive functions (Test for Memory 10 words [15], Wisconsin Card Sorting Test (WCST) [16], Trail making test (TMT) Part A and B [17], Verbal fluency test [18]) were carried out. Assessments occurred at baseline and 3, 6, and 12 months.

### Statistical analysis

Comparison of independent variables was carried out with the Mann-Whitney U test; comparison-dependent variables were assessed using the Wilcoxon matched-pair test and Friedman analysis of variance (ANOVA). Spearman rank order correlation (*r *values) was used as a measure of association, including baseline patient characteristics and achievement of remission in the 1-year observational study. The predictive value of various factors in the population study was assessed with logistic regression. The general linear model (univariate) procedure was used to perform analysis of covariance (ANCOVA) to control for the effects of positive, negative, and general PANSS scores (covariates) on symptomatic remission achievement (dependent variable), with different ICD-10 diagnoses (F20.00, F20.01, F20.02, F20.3, F20.5, F20.6) as a categorical factor. Odds ratios (OR) were calculated for associations between episodic or chronic courses and symptomatic remission in the RLAI group. Missing data were completed with single sample averages in the cross-sectional population study and last observation carried forward (LOCF) in the 6-month follow-up and 1-year observational studies.

### Ethical considerations

The treatment regime and design of the study were approved by the local ethical committee of the Moscow Research Institute of Psychiatry and comply with principles of the Helsinki Declaration. All included patients gave informed consent to participate in this study.

## Results

### Cross-sectional study

#### Sample characteristics

A total of 233 outpatients in 2 psychiatric healthcare districts had diagnoses of schizophrenia and schizoaffective disorder (F20 and F25), but only 203 of them gave agreement for participation and were thus included in the cross-sectional study (114 in the first district and 89 in the second district). A total of 114 (56.2%) were women. The average age was 52.8 years (SD, 15.0), the average illness duration 24.4 years (SD, 13.2). Most patients (144 (70.9%)) had a diagnosis of paranoid schizophrenia (F20.0); 2 (1.0%) had hebephrenic schizophrenia (F20.1), 4 (2.0%) catatonic schizophrenia (F20.2), 10 (4.9%) undifferentiated schizophrenia (F20.3), 28 (13.8%) residual schizophrenia (F20.5), 6 (3.0%) simple schizophrenia (F20.6) and 9 (4.4%) schizoaffective disorder (F25). The mean PANSS total score was 69.2 (SD, 24.9) and the GAF score was 56.7 (SD, 11.0).

#### Pharmacoepidemiological data

Among the 203 outpatients included in the analysis, 126 (62%) were treated with first-generation antipsychotics (including combined therapy), 25 (12%) with second-generation antipsychotics (including combined therapy), 14 (7%) took a combination (first-generation and second-generation antipsychotics) and 38 (19%) did not receive antipsychotic treatment. There were no significant differences in remission rate, total PANSS score, or GAF score between groups of first-generation or second-generation antipsychotics observed. However, the average age and duration of treatment were lower in the group of patients treated with atypical antipsychotics: mean age 35.7 years (SD, 9.1) and 54.6 years (SD, 10.4), respectively; duration of treatment 8.2 years (SD, 5.9) and 24.3 years (SD, 12.5), respectively (Mann-Whitney, *P *< 0.001).

#### Cross-sectional study with a 6-month follow-up period

The distribution of patients was as follows: 64 (31.5%) out of 203 patients met the symptomatic criterion, whereas 139 (68.5%) did not. After the 6-month follow-up period for assessment of symptomatic stability, 53 (82.8%) out of 64 patients maintained remission. Only 105 (75.5%) of 139 patients who did not meet the symptomatic criterion in the cross-sectional study were stable over 6 months.

The mean GAF score was significantly higher in the group with full remission (72.5 (SD, 7.5)) than in the stable group that did not meet both criteria (51.8 (SD, 8.7)) (Mann-Whitney, *P *< 0.05).

Most patients who met full remission criteria had diagnoses of episodic (21 (39.6%)) and remittent (8 (15.0%)) courses of paranoid schizophrenia or schizoaffective disorder (9 (17.0%)). The majority of patients with an episodic course of disorder were classified as episodic with progressive deficit (71.4%). In the group of stable patients who did not meet symptomatic remission criteria, more severe diagnoses prevailed: continuous (43 (41.0%)) and episodic (31 (29.5%)) (primarily with stable deficit) courses of paranoid schizophrenia, undifferentiated (6 (5.7%)) and residual (16 (15.2%)) schizophrenia, hebephrenic schizophrenia (2 (1.9%)), and catatonic schizophrenia (3 (2.9%)) (Table 1).

**Table 1:** Distribution of stable patients according to *International Classification of Diseases*, 10th edition (ICD-10) diagnosis and the achievement of remission criteria (*n *= 158)

ICD-10 diagnosis	Group of stable patients who maintained symptomatic remission for 6 months (53 patients/21.6% of 203)	Group of patients who were stable for 6 months, but did not satisfy the symptomatic remission criteria (105 patients/51.7% of 203)
Paranoid schizophrenia:		
Continuous course	2 (3.8%)	43 (41.0%)
Episodic course with progressive and stable deficit	21 (39.6%)	31 (29.5%)
Episodic remittent course	8 (15.0%)	0
Hebephrenic schizophrenia	0	2 (1.9%)
Catatonic schizophrenia	0	3 (2.9%)
Undifferentiated schizophrenia	2 (3.8%%)	6 (5.7%)
Residual schizophrenia	9 (17.0%)	16 (15.2%)
Simple schizophrenia	2 (3.8%)	4 (3.8%)
Schizoaffective disorder	9 (17.0%)	0

Analysis of each of the eight core remission PANSS symptoms indicated that stable patients could be characterized by a specific threshold level according to their ICD-10 diagnosis (patients with catatonic and hebephrenic schizophrenia were excluded because they were an unrepresentative sample). First, the proposed threshold was achievable for all symptoms only for those with paranoid schizophrenia with a remittent course and schizoaffective disorder. Second, patients with different clinical types of schizophrenia failed to meet this level in different symptomatic dimensions. For example, those with paranoid schizophrenia with a continuous course and undifferentiated schizophrenia did not meet symptomatic remission for positive and negative symptom dimensions, while those with paranoid schizophrenia with an episodic course, residual schizophrenia, and simple schizophrenia did not meet the negative symptom dimension. More detailed information is shown in Table 2.

**Table 2:** Threshold level for each of remission criteria symptoms in a group of stable patients according to *International Classification of Diseases*, 10th edition (ICD-10) diagnosis (*n *= 158)

Dimension of psychopathology according to three-factor model of schizophrenia	ICD-10 diagnosis (PANSS symptoms)	Paranoid schizophrenia, continuous course	Paranoid schizophrenia, episodic course	Paranoid schizophrenia, remittent course	Undifferentiated schizophrenia	Residual schizophrenia	Simple schizophrenia	Schizoaffective disorder
Psychoticism (reality distortion)	Delusion	3.7 (± 1.0)/5	2.5 (± 1.1)/3	1.3 (± 0.5)/1.5	3.7 (± 0.9)/4	1.7 (± 0.9)/2	1.0 (± 0)/1	1.0 (± 0)/1
	Unusual thought content	3.8 (± 1.1)/5	2.3 (± 1.1)/3	1.5 (± 0.5)/2	3.0 (± 1.0)/4	1.7 (± 1.0)/3	1.5 (± 0.8)/2	1.2 (± 0.7)/1
	Hallucinatory behavior	3.5 (± 1.2)/4	2.0 (± 1.0)/3	1.1 (± 0.4)/1	3.7 (± 0.7)/4	1.6 (± 0.7)/2	1.0 (± 0)/1	1.0 (± 0)/1
Disorganization	Conceptual disorganization	2.2 (± 1.2)/3	1.8 (± 0.9)/3	1.1 (± 0.4)/1	2.3 (± 1.0)/3	1.9 (± 0.8)/3	1.2 (± 0.4)/1	1.1 (± 0.3)/1
	Mannerisms/posturing	2.2 (± 1.3)/3	1.9 (± 1.3)/3	1.0 (± 0)/1	1.9 (± 1.2)/3	2.0 (± 0.9)/3	1.8 (± 1.0)/3	1.0 (± 0)/1
Negative symptoms (psychomotor poverty)	Blunted affect	3.9 (± 0.9)/4	3.2 (± 0.9)/4	1.3 (± 0.5)/1.5	3.5 (± 0.8)/4	3.2 (± 1.0)/4	4.0 (± 0.6)/4	1.6 (± 0.7)/2
	Social withdrawal	4.3 (± 1.0)/5	2.9 (± 1.3)/4	1.8 (± 0.7)/2	3.2 (± 1.4)/4	3.3 (± 0.9)/4	3.8 (± 0.8)/4	1.8 (± 0.7)/2
	Lack of spontaneity	4.0 (± 1.0)/5	2.5 (± 0.9)/3	1.4 (± 0.7)/1.5	3.3 (± 1.2)/4	3.0 (± 0.8)/4	3.9 (± 0.7)/4	1.0 (± 0)/1

Values are mean, standard deviation (± SD)/75th percentile unless otherwise stated.

PANSS = Positive and Negative Syndrome Scale.

For the logistic regression, the dependent variable was symptomatic remission, while ICD-10 diagnosis (F20.01, F20.03, F25 vs other), age, illness duration, first-degree relatives with psychotic disorder, disability, previous antipsychotic treatment and GAF score were used as independent variables. The analysis indicated that ICD-10 diagnosis (F20.01, F20.03, F25 vs other) (OR = 5.95) and GAF score (OR = 1.29) predicted the outcome of symptomatic remission, whereas history of psychotic disorder in first-degree relatives (OR = 0.44) and presence of disability (OR = 0.64) decreased the likelihood of symptomatic remission (Table 3). Age, illness duration, and previous antipsychotic treatment (atypical vs typical) variables did not reach statistical significance.

**Table 3:** Odds ratios and 95% confidence intervals of the factors significantly predicting symptomatic remission

	b	Odds ratio (OR)	95% Confidence interval	*P *value
ICD-10 diagnosis (F20.01, F20.03, F25 vs other)	1.78	5.95	1.95 to 18.10	< 0.001
First degree relatives with psychotic disorder	-0.85	0.44	0.18 to 0.84	< 0.05
Disability (yes or no)	-1.4	0.64	0.13 to 0.49	< 0.001
GAF score	0.29	1.29	1.23 to 1.46	< 0.001

GAF = Global Assessment of Functioning; ICD-10 = *International Classification of Diseases*, 10th edition.

### Observational therapeutic study

#### Sample characteristics

In all, 77 subjects (42 patients receiving RLAI and 35 patients receiving routine treatment) were recruited to the observational therapeutic study. At baseline, none of the patients met the symptom severity component of the proposed remission criteria, but they were stable for 6 consecutive months. Most patients were women in the RLAI (24 (57.1%)) and routine treatment (19 (54.3%)) groups. The average age was 43.7 years (SD, 13.4) in the RLAI group and 45.4 years (SD, 14.2) in the routine treatment group; previous treatment duration was 16.8 years (SD, 11.7) and 15.7 years (SD, 12.3), respectively. In the RLAI group, most patients had diagnoses of paranoid schizophrenia with continuous (15 (35.7%)) and episodic (15 (35.7%)) courses. Other diagnoses were catatonic (1 (2.4%)), undifferentiated (3 (7.1%)), residual (5 (11.9%)) and simple schizophrenia (3 (7.1%)). In the routine treatment group, paranoid schizophrenia with continuous (11 (31.4%)) and episodic (13 (37.1%)) courses also predominated over other diagnoses. Other diagnoses were catatonic schizophrenia (1 (2.9%)) and undifferentiated (2 (5.7%)), residual (6 (17.1%)), and simple (2 (5.7%)) schizophrenia. There were no significant differences between therapeutic groups (Table 4).

**Table 4:** Demographics and baseline characteristics in the 1-year observational therapeutic study

Characteristics	Risperidone (long-acting, injectable) (RLAI) group	Routine treatment group
Number of patients	42	35
Sex: male/female	18/24	16/19
Age (SD)	43.7 (13.4)	45.4 (14.2)
Duration of disease (SD)	16.8 (11.7)	15.7 (12.3)
Number of previous hospitalizations (SD)	4.7 (2.5)	4.2 (2.8)
Paranoid schizophrenia:		
Continuous course	15/35.7%	11/31.4%
Episodic course with progressive and stable deficit	15/35.7%	13/37.1%
Remittent course	0	0
Hebephrenic schizophrenia	0	0
Catatonic schizophrenia	1/2.4%	1/2.9%
Undifferentiated schizophrenia	3/7.1%	2/5.7%
Residual schizophrenia	5/11.9%	6/17.1%
Simple schizophrenia	3/7.1%	2/5.7%
Schizoaffective disorder	0	0
PANSS total score (SD)	66.1 (10.7)	68.7 (12.3)
PANSS positive score (items 1 to 7) (SD)	14.5 (4.5)	16.0 (5.1)
PANSS negative score (items 8 to 14) (SD)	18.8 (4.3)	19.1 (4.8)
PANSS general score (items 15 to 30) (SD)	33.1 (5.4)	35.3 (6.8)
Simpson-Angus scale score (SD)	4.9 (6.3)	5.1 (6.1)
PSP score (SD)	52.0 (12.3)	51.3 (13.1)
ROMI score		
Compliance subscale	14.9 (2.3)	15.4 (2.6)
Non-compliance subscale	17.1 (3.3)	16.4 (4.1)

No significant differences between groups.

PANSS = Positive and Negative Syndrome Scale; PSP = Personal and Social Performance; ROMI = Rating of Medication Influences.

#### Achievement of remission

**Figure 1:**
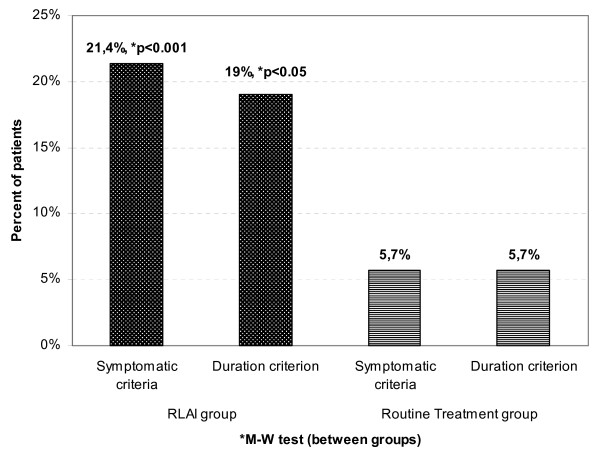
**Remission rates at the end point (12 months) in the risperidone (long-acting, injectable) (RLAI) and control groups (last observation carried forward used for missing data)**.

The symptomatic criterion in the RLAI group was met by 16.7% of patients at month 3, 23.8% at month 6 and 21.4% at month 12 (Friedman ANOVA, *P *< 0.0001). However, only 19% of patients met both the symptom and duration criteria (Friedman ANOVA, *P *< 0.0001). In the routine treatment group, only 5.7% of subjects achieved full remission at month 12 (Friedman ANOVA, *P *= 0.03). Starting from month 3, there was a significant difference between the groups (Mann-Whitney, *P *< 0.05) that continued to months 6 and 12 (Mann-Whitney, *P *< 0.0001) (Figure 1).

#### Scores for symptom change

There was a statistically significant decrease at end point for total PANSS score of 13.8% in the RLAI group (66.1 (SD, 10.7) and 57.0 (SD, 13.7), respectively; *P *< 0.0001) and 6.8% in the routine treatment group (68.7 (SD, 12.3) and 64.0 (SD, 13.4), respectively; *P *< 0.05); the difference between the groups was also significant (Mann-Whitney, *P *< 0.001). The reduction in positive PANSS score was 10.3% in the RLAI group (14.5 (SD, 4.5) and 13.0 (SD, 5.3), respectively; *P *< 0.05) and 7.5% in the routine treatment group (16.0 (SD, 5.1) and 14.8 (SD, 5.5), respectively), a significant between-group effect (Mann-Whitney, *P *< 0.001). The negative PANSS score reduction was greater in the RLAI group, 10.1% (18.8 (SD, 4.3) and 16.9 (SD, 4.8), respectively; *P *< 0.001), as opposed to only 2.1% in the routine treatment group (19.1 (SD, 4.8) and 18.7 (SD, 5.6), respectively), a significant between-group effect (Mann-Whitney, *P *< 0.05). Furthermore, there was a significant decrease in the general PANSS score in the RLAI group, 18.1% (33.1 (SD, 5.4) and 27.1 (SD, 7.0), respectively; *P *< 0.0001), and in the routine treatment group, 5.9% (19.1 (SD, 4.8) and 18.7 (SD, 5.6), respectively; *P *< 0.05) (Mann-Whitney, *P *< 0.0001) (Table 5).

**Table 5:** Changes of PANSS, PSP and ROMI total and subscales score over the 1-year observational therapeutic study

PANSS score	Risperidone (long-acting, injectable) (RLAI) group, n = 42	Routine treatment group, n = 35	Mann-Whitney test (between groups)
PANSS total score (SD)			
1 day	66.1 (10.7)	68.7 (12.3)	-
12 month	57.0 (13.8) ***	64.0 (13.4)*	< 0.01
PANSS positive score (items 1 to 7) (SD)			
1 day	14.5 (4.5)	16.0 (5.1)	-
12 month	13.0 (5.3)*	14.8 (5.5)	< 0.01
PANSS negative score (items 8 to 14) (SD)			
1 day	18.8 (4.3)	19.1 (4.8)	-
12 month	16.9 (4.8)**	18.7 (5.6)	< 0.05
PANSS general score (items 15 to 30) (SD)			
1 day	33.1 (5.4)	35.3 (5.4)	-
12 month	27.1 (7.0)***	33.2 (6.8)*	< 0.001
PSP score (SD)			
1 day	52.0 (12.3)	51,3 (13.1)	-
12 month	60.1 (12.3)***	53.6 (13.5)*	< 0.0001
ROMI, compliance subscale score (SD)			
1 day	14.9 (2.3)	15.7 (3.0)	-
12 month	15.2 (2.5)	15.5 (2.9)	-
ROMI, non-compliance subscale score (SD)			
1 day	17.1 (3.3)	16.4 (4.1)	-
12 month	15.3 (3.4)***	16.0 (2.9)	< 0.05

Wilcoxon test (baseline vs end point): *P < 0.05; **P < 0.01; ***P < 0.001.

PANSS = Positive and Negative Syndrome Scale; PSP = Personal and Social Performance; ROMI = Rating of Medication Influences.

#### Functioning and compliance

A significant improvement was observed in the end point PSP scores in the RLAI group, 15.6% (52.0 (SD, 12.3) and 60.1 (SD, 12.3), respectively; *P *< 0.0001), while there was a lower increase of 4.5% in the routine treatment group (51.3 (SD, 13.1) and 53.6 (SD, 13.5), respectively; *P *< 0.0001), with a significant between-group effect (Mann-Whitney, *P *< 0.0001). In the patients who met remission criteria (pooled two-group analysis) the mean PSP score was 73.8 (SD, 12.4,) whereas it was 56.6 in patients who did not meet the remission criteria (SD, 12.0) (Mann-Whitney, *P *< 0.001).

There was no significant improvement in the ROMI compliance subscale in either group. However, there was a significant decrease in the non-compliance subscale score for the RLAI group of 10.5% (17.1 (SD, 3.3) and 15.3 (SD, 3.4), respectively; *P *< 0.0001), compared with only 2.4% for the routine treatment group (16.4 (SD, 4.1) and 16.0 (SD, 2.9), respectively) (Mann-Whitney, *P *< 0.05).

#### Safety

In the RLAI group, there was a 71.4% reduction in the SAS score to end point (4.9 (SD, 6.3) and 1.4 (SD, 1.9), respectively; *P *< 0.0001); in the routine treatment group SAS score reduction was lower (13.7% (5.1 (SD, 6.1) and 4.4 (SD, 5.4), respectively)). There were ten patients with weight gain more that 7% over the 1-year study in the RLAI group, and three in the routine treatment group. In the RLAI group, secondary amenorrhea occurred in nine patients, galactorrhea in one, menorrhagia that caused discontinuation of the study and further hospitalization in one, headache in two, dizziness in three and stuffiness in the nose in two. In the routine treatment group, secondary amenorrhea was observed in two (one patient taking oral risperidone and one taking haloperidol decanoate and chlorpromazine), dizziness in three (taking oral haloperidol, zuclopenthixol decanoate, and oral haloperidol with chlorpromazine), constipation in two (one taking oral zuclopenthixol, one taking chlorpromazine), and insomnia in one (flupentixol decanoate).

#### Factors associated with achievement of symptomatic remission

A significant correlation (*P *< 0.05) was found between symptomatic remission and PANSS total score (*r *= -0.61), plus with positive (*r *= -0.57), negative (*r *= -0.39), and general (*r *= -0.61) PANSS scores. Symptomatic remission was associated with the results of cognitive tests: WCST (*r *= -0.38), TMT Part B (*r *= -0.42), and PSP score (*r *= 0.48), as well as with subscales of the SF-36 such as physical functioning (*r *= 0.42) and social functioning (*r *= 0.47).

**Figure 2:**
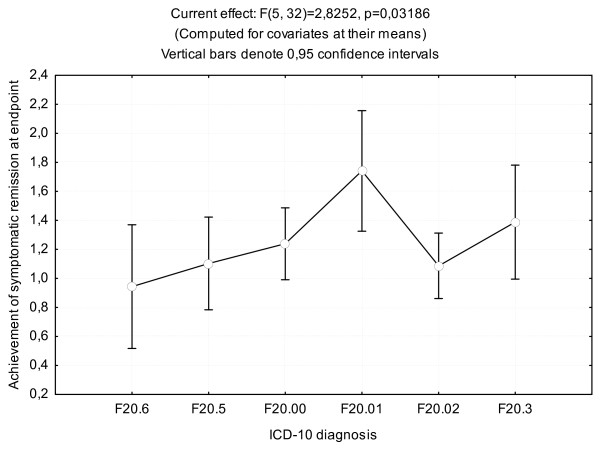
**Symptomatic remission achievement in the risperidone (long-acting, injectable) (RLAI) group according to *International Classification of Diseases*, 10th edition (ICD-10) diagnosis (analysis of covariance (ANCOVA))**.

ANCOVA in the RLAI group found that ICD-10 diagnosis was statistically significant for achievement of symptomatic remission (Figure 2). The adjusted *r*^2 ^value for this model was 0.38 (*F *= 4.002271, *P *= 0.002204). Overall, patients with paranoid schizophrenia and progressive deficit (F20.01) had a greater possibility of achieving symptomatic criterion of remission than patients with other diagnoses. Moreover, seven (four with F20.01, two with F20.02, one with F20.3) of the nine patients who met symptomatic remission criterion in the RLAI group had an episodic course of schizophrenia, and the OR for symptomatic remission was 4.0 in patients with episodic schizophrenia compared with a chronic course of schizophrenia.

## Discussion

The prevalence of symptomatic remission in schizophrenia by Andreasen criteria vary widely across reported studies (17% to 88%) [19,20]. However, for most samples in cross-sectional studies in naturalistic settings approximately one-third of individuals had symptomatic remission [6,21,22]. These results were confirmed in our cross-sectional study in an outpatient schizophrenia population in one territorial sector of Moscow city, where 31.5% of patients met the symptomatic remission criterion. It has to be noted that 62% of patients were treated with a first-generation antipsychotic and only 12% with a second-generation antipsychotic. In contrast, a Spanish cross-sectional study using 80.1% second-generation antipsychotics, the rate of symptomatic remission was 44.8% (452 of 1,010 patients) [23]. Maintenance of remission status over a 6-month period without changes to current antipsychotic treatment constituted 82.8% of patients, which is very close to that reported in the previous study with the same follow-up duration [23]. But for total outpatient population full remission criteria were only fulfilled in 26.1% of cases. In a recent German naturalistic study the remission rate over the 1-year follow-up period was even less: 10.3% of patients [24].

The most significant factors predicting achievement of remission reported by Lambert *et al*. [25], using multivariate regression analysis of 12 studies, are (i) shorter duration of untreated psychosis, (ii) better premorbid adjustment, (iii) lower psychopathology or illness severity scores at baseline, (iv) better functioning level at baseline, (v) early improvement in symptoms or functioning, and (vi) medication adherence during treatment. These predicting factors were supported by our observational study. The most important baseline patient characteristics associated with symptomatic remission were symptom severity (PANSS score at baseline), executive functions (WCST, TMT Part B), social and personal functioning (PSP score), and quality of life (physical functioning and social functioning subscales of the SF-36). The relationship between symptomatic remission and cognitive improvement was also confirmed by Buckley *et al*. [26]. Additionally, we have observed that diagnoses of episodic (with progressive deficit) and remittent courses of schizophrenia were associated with an increased chance of remission as compared to continuous and episodic courses with stable deficit. This observation was also noted previously by Wobrock *et al*. [7]. They reported that outpatients with different types of schizophrenia by ICD-10 definition had differing chances of achieving symptomatic remission: patients with paranoid schizophrenia had a greater possibility of meeting the international remission criteria than those with other diagnoses, particularly residual schizophrenia. Furthermore, each of the ICD-10 types and courses of schizophrenia in our study differed in the threshold for the eight core PANSS items in our sample of stable patients. When Eberhard *et al*. [27] analyzed a 5-year risperidone trial, they found that the core eight PANSS items are quite common in patients with schizophrenia and non-schizophrenic disorders with psychotic symptoms. However, discriminate analysis has highlighted G6 (depression) and G15 (preoccupation) as potentially important symptoms for differentiating other psychotic disorders from schizophrenia. Therefore, our opinion is that the symptom threshold is very strict for chronic patients with schizophrenia and it is necessary to develop specific remission criteria for different clinical forms and courses of schizophrenia. This point of view agrees with previous research performed in Russia using a categorical approach to the psychopathology of schizophrenia and defining remission within an interval from full recovery (symptomatic and functional) to marked deficit with long-term symptomatic stability of patients [28,29]. This definition is partly supported by findings in our study: 105 (75.5%) of 139 patients who did not meet the symptomatic criterion in the cross-sectional study were stable over 6 months. Moreover, ANCOVA in the RLAI group in our observational study showed that ICD-10 diagnosis was statistically significant for achievement of symptomatic remission. Schizophrenia is heterogeneous in psychopathology and its outcomes, and it is impossible to ignore the different clinical types of the disorder. It is not surprising that patients with schizoaffective disorder and remittent or episodic courses of paranoid schizophrenia had a greater chance to achieve symptomatic remission. In the observational 1-year study, stable patients switched to RLAI had a significant reduction of psychopathological symptoms and non-compliance rate, as well as an improvement in social and personal functioning; however, only 21.4% of patients met symptomatic remission criterion and only 19% achieved full remission. In the routine treatment group, the remission rate at end point was much lower, at 5.7%. This finding agrees with some previous studies. Lasser *et al*. [30] found that 82 (20.8%) of the 394 stable patients who did not meet symptomatic remission criterion at baseline achieved it over 1 year of treatment with RLAI. However, Rossi *et al*. [31] reported a higher level of sustained remission; 32% of a sample of 347 stable patients that were switched to RLAI met remission at week 52. Generally, patients considered to be stable may not be at their optimal symptomatic and functional levels, and modern therapeutic approaches can improve their outcomes. However, the proportion of stable patients who did not meet remission in all studies is remarkable, and for most chronic patients the symptomatic threshold is unachievable. This statement is also supported by results from the medication phases of the Clinical Antipsychotic Trials of Intervention Effectiveness (CATIE) study. Only 11.7% of patients attained symptomatic remission and then maintained it for at least 6 months, and 55.5% experienced no symptom remission at any stage [32].

There are limitations to this study. First of all, this is one-site study, but despite this fact as patients from the outpatient psychiatric service in two healthcare districts of a big city were chosen randomly, we believe it can be representative; future research in Russia is necessary however, featuring multicenter studies with sites in different regions, including rural ones. Secondly, the sample size was not specially estimated for the exploratory objectives, and some analyses only included a small number of subjects. Thirdly, the therapeutic study was observational in a naturalistic setting and did not assume randomization or a blinded design; nevertheless the clinical and demographic characteristics of the two district populations of stable patients were comparable. However, the psychotherapeutic influence of switching to a completely new therapeutic approach and additional attention from medical staff with visits every 2 weeks for injections could be important. Therefore, it is necessary to interpret our results cautiously, and obviously further research is needed to confirm our findings and the importance of revising the remission criteria according to the clinical courses and types of schizophrenia and patient functioning level.

## Conclusions

Our study demonstrates lower remission rates among outpatients with schizophrenia and schizoaffective disorder treated mainly with first-generation antipsychotics, and the possibility to improve remission rate by switching to long-acting atypical antipsychotics. However, the symptomatic criterion of remission suggested by the Remission in Schizophrenia Working Group [3] was unachievable for most stable patients in spite of the significant reduction of their symptoms. In addition, these criteria ignore the clinical course of schizophrenia and the functioning level, which are of great importance for the remission state, so it seems clinically relevant to develop revised remission criteria that refer to the heterogeneity of this disorder and to the variety of social outcomes.

## Competing interests

The authors declare that they have no competing interests.

## Authors' contributions

SNM provided the idea and design of the study, participated in the study organization, patient consulting, and data analysis and drafting of the manuscript. AVP performed clinical assessments including the scale rating and cognitive testing, performed the statistical analysis and wrote the text. UVU, as a district psychiatrist in Moscow Psychiatric Outpatient Service #21, was responsible for the recruitment, diagnosing and treatment of the patients. All authors read and approved the final manuscript.
